# The effect of improving solid waste collection on waste disposal behaviour and exposure to environmental risk factors in urban low‐income communities in Pakistan

**DOI:** 10.1111/tmi.13787

**Published:** 2022-06-21

**Authors:** Wolf‐Peter Schmidt, Irfan Haider, Musarat Hussain, Mahpara Safdar, Farooq Mustafa, Terrill Massey, Gerald Angelo, Mari Williams, Richard Gower, Zoone Hasan, Hugh Sharma Waddington, Nomana Anjum, Adam Biran

**Affiliations:** ^1^ Department of Disease Control London School of Hygiene and Tropical Medicine London UK; ^2^ Department of Environmental Design, Health and Nutritional Sciences Allama Iqbal Open University Islamabad Pakistan; ^3^ Tearfund, Pakistan Office Islamabad Pakistan; ^4^ Tearfund UK Office London UK; ^5^ Pakistan Mission Society, PMS Islamabad Pakistan

**Keywords:** public health, recycling, synanthropic flies, waste disposal

## Abstract

**Objective:**

To estimate the effect of improving waste collection services on waste disposal behaviour and exposure to environmental risk factors in urban, low‐income communities in Pakistan.

**Methods:**

We enrolled six low‐income communities in Islamabad (Pakistan), four of which received an intervention consisting of a door‐to‐door low‐cost waste collection service with centralised waste processing and recycling sites. Intervention communities underwent community‐level and household‐level mobilisation. The effect of the intervention on waste disposal behaviour, exposure to waste and synanthropic fly counts was measured using two cross‐sectional surveys in 180 households per community.

**Results:**

Intervention communities had less favourable socio‐economic indicators and poorer access to waste disposal services at baseline than control communities. Use of any waste collection service increased from 5% to 49% in the intervention communities (difference 44%, 95% CI 41%, 48%), but the increase was largely confined to two communities where post‐intervention coverage exceeded 80% and 90%, respectively. An increase in the use of waste collection services was also found in the two control communities (from 21% to 67%, difference 47%, 95% CI 41%, 53%). Fly counts decreased by about 60% in the intervention communities (rate ratio 0.4, 95% CI 0.3, 0.4) but not in the control communities (rate ratio 1.52, 95% CI 1.1, 2.2). The decrease in fly counts was largely confined to the two high‐coverage intervention communities.

**Conclusion:**

Introduction of a low‐cost waste collection service has the potential for high uptake in low‐income communities and for decreasing the exposure to waste and synanthropic flies at household level. Intervention success was constrained by low uptake in half of the intervention communities.

## INTRODUCTION

Solid waste or refuse is generated by households, agriculture, industry and by institutions such as schools, offices and medical facilities. Management of that waste is a major industry and livelihood source globally. Its mismanagement is likely to be an important factor underlying the global burden of disease [1]. Economic development and urbanisation go hand‐in‐hand with increased waste, which becomes a more pressing problem as countries transition from low‐ to middle‐ and high‐income status. Solid waste management targets are included under Sustainable Development Goals 11 and 12 [2]. The World Bank has estimated that urban residents generate 1.2 kg of municipal solid waste per person per day [3], resulting in 2 billion tons per year [4]. Waste per person‐day is three times larger in cities in the richest countries than the poorest, with the same variation at different income levels within countries [5]. Some research has been conducted into the effects of solid waste management on public health, and on sanitary workers such as formal waste collection workers and informal waste pickers. It suggests health impacts both on those working with waste [6], and on communities in the vicinity of waste disposal sites [7], particularly in low‐income settings [1, 3].

However, a large proportion of the household solid waste that is generated is never collected by any formal system of waste management. An estimated 2 billion people lack access to solid waste collection and 3 billion people lack access to controlled waste disposal [8]. The lowest collection rates are in Africa and Asia, with estimated collection rates of 25%–70% and 50%–90%, respectively [8].

Inappropriately managed solid waste disposal is likely to be associated with disease and environmental pollution, bringing social and economic costs. Mosquitoes breed in stagnant water in discarded tin cans or flooding caused by waste‐blocked drainage ditches, propagating malaria, dengue and zika virus; flies and cockroaches breeding in open waste may spread gastro‐intestinal pathogens causing diarrhoea [1]; exposure to human and animal faeces in waste could be associated with helminth infection [1]; rats living on domestic waste may lead to outbreaks of leptospirosis [9]. Burning of solid waste around homes and in informal dumpsites has been estimated to contribute to 270,000 deaths per year due to ailments including lung cancer and heart disease, of which 191,000 deaths would be prevented if informal waste burning were stopped [10]. Various plastic additives and heavy metals have been identified in solid waste leachate at dumpsites, and it may be assumed that the same compounds will contaminate informal dumpsites. One of the most widespread hazards is lead. Lead‐containing wastes include cement, paint, vehicles (e.g. lead‐acid batteries), fertiliser, compost, and general household waste. The IHME Global Burden of Disease study estimates over 500,000 deaths worldwide annually attributable to lead exposure [11]. Apart from specific health effects, poorly managed solid waste may also affect general wellbeing and social status, for example, by stigmatising communities that are perceived as undesirable due to the closeness to informal waste disposal sites. Overall, there is a strong case for improving waste disposal practices not only in high‐income settings (which produce a disproportionate amount of waste), but also among low‐income populations who may have fewer opportunities to avoid exposure to adverse health effects of poorly managed waste.

The Saaf Mahol (“Clean Environment”) project in Islamabad, Pakistan, introduced daily waste collection in urban, low‐income communities that previously had only informal and unsystematic waste collection services. The project presented an opportunity to study the reach and effect of improved waste collection services, and to further our understanding of the links between solid waste and health in urban low‐income settings.

The aim of this study was to estimate the effect of introducing systematic solid waste management in a poor urban environment on waste disposal practices, disease vectors and markers of environmental exposure in the target population.

## METHODS

### Study design and study population

**FIGURE 1:**
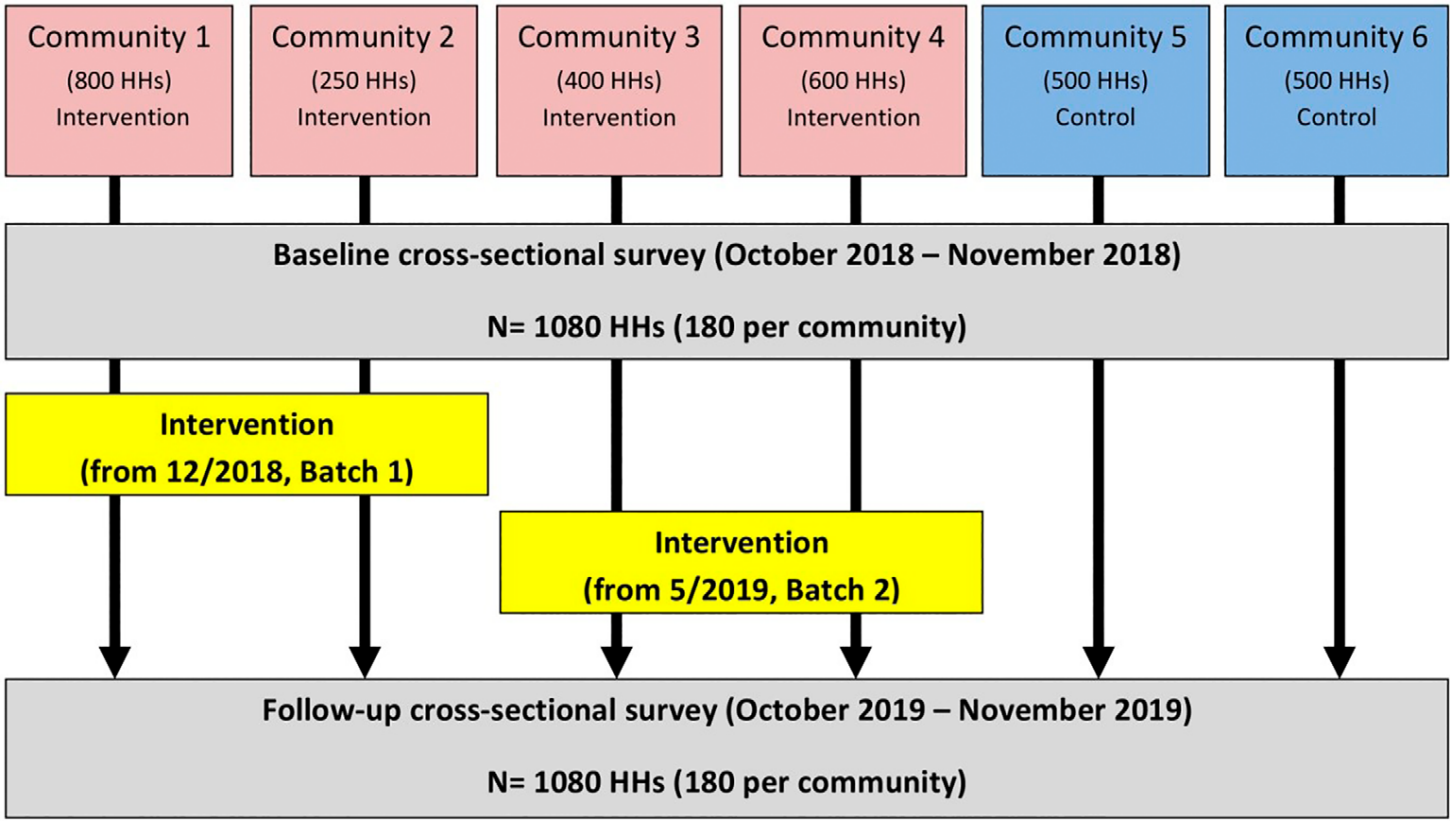
Study flow diagram

We used a before‐and‐after study design, collecting data through cross‐sectional surveys at baseline and follow‐up. The study was carried out in six of the seven urban, low‐income communities in Islamabad, Pakistan, where the Pakistan Mission Society (PMS, the implementing partner) was operating. Four of these communities received a waste management intervention while the two remaining communities served as controls. The study communities were demarcated by large roads, rivers and fields which separated them from surrounding neighbourhoods. The communities were informal or semi‐formal settlements with some variation in the materials used for housing as well as in access to services such as water, sanitation and electricity. The four intervention communities were chosen based on the perceived need for improving waste disposal, in particular, absence of existing for services. The choice of the two control communities was limited, as only three eligible control communities were accessible to the research team. Among these three, two were chosen for their greater similarity to the intervention communities in terms of socio‐economic development. However, these were not ideal controls as both were more established, having better legal status and better access to public services such as water, electricity and waste disposal than the intervention communities. The size of each community ranged between 230 and 800 households (Figure 1).

Baseline and follow‐up surveys were conducted in 1080 households (180 households per community). Both surveys used the same methods and questionnaire tool. Households were enrolled by systematic sampling of every *n*th household, with *n* being determined by dividing the population size in a community by 180, ignoring decimals. Enrolment started at the edge of each community using the same starting points at baseline and follow‐up, with the first household to be enrolled chosen among the first *n* households at random. If a household refused to participate or was absent, the next household was selected. No efforts were made to enrol the same households at baseline and follow up. This avoided the need to collect personal details, which people were reluctant to disclose and allowed households to remain anonymous. In practice, given the large sampling fraction in smaller communities, some households were likely to be enrolled at both time points.

### Intervention

The SAAF Mahol project, implemented by Tearfund partner Pakistan Mission Society and funded by Tearfund, aimed to replace existing, informal waste disposal practices with organised collection and separation of waste for recycling or reuse. The intervention had three elements: (1) awareness‐raising activities at community and household level; (2) a regular, subscription‐based, doorstep waste collection service by staff members who receive a wage by PMS; (3) the creation and operation of recycling sites where waste was separated into organic matter (comprising about 70% of the volume, which was composted for sale as a soil improver) and non‐organic recyclables (plastics, paper and metals) which were sold to local waste dealers who sold it on for recycling. The revenues generated were used to help cover the cost of the project. The involvement of local waste dealers was intended to prevent them from experiencing a loss of income resulting from the project.

The intervention was delivered to communities in two batches (Figure 1). Two communities considered by the implementing organisation to be those with greatest need for waste disposal improvement (“Batch 1”) received the intervention from December 2018. The other two communities (“Batch 2”) received the intervention from May 2019. The intervention was implemented with the aim of establishing an ongoing service with no specified end date.

### Outcome assessment

Outcome indicators were assessed at baseline and follow‐up (10 months or 5 months after the start of implementation). Baseline data were collected in October and November 2018, with the follow‐up survey done in October and November 2019. This was to control for possible seasonal variation in outcomes, particularly fly numbers.

Data were collected by field workers who had not been involved in intervention implementation. Data collection methods included a household questionnaire, fly‐counts in cooking areas and a survey of community waste dumping and burning sites. The questionnaire covered socio‐economic variables, current waste disposal practices, satisfaction with current waste disposal practices, perceived exposure to waste, perceived exposure to smoke from burning of waste, frequency of sighting of rodents in the home, and frequency of sighting of cockroaches in the home. The questionnaire at baseline and follow‐up was identical.

The numbers of flies present near the cooking area were assessed using blue sticky tape traps [12], either indoors or outdoors, depending on the location of the cooking area. The tape was cut to a size of 150 mm × 245 mm and hung from the ceiling or from an electric cable at a height of between 1 and 1.5 m. The tape was collected on the next day. Flies were counted immediately on collection, before discarding the tape. Fly counts were disaggregated by genus; *musca* and *calliphoridae*. Other insects caught on the tapes were ignored.

At baseline and follow up, each neighbourhood was systematically surveyed for informal waste sites by Master students from Allama Iqbal Open University. The area of waste was measured using tape measures and recorded in square meters. Attempts to measure the volume proved too difficult. Evidence of waste burning was also recorded. Burn sites were recorded as numbers, not as square meters, as their size was usually small. Often several burn sites would be observed at a single informal waste disposal site. At follow‐up, the team revisited the previously identified sites to assess any changes in status since the baseline, before enumerating any newly identified sites.

### Sample size and statistical analysis

The sample size calculation was driven by expected fly numbers, as these were deemed to be highly variable across communities. Based on data from an urban, low‐income setting in India [13], we assumed that the mean log fly count caught in 24 h would be around 2.6 (SD 1.7). We assumed the intervention would reduce this figure to 2.3, which resulted in a sample size of 505 households to be sampled before and after the intervention in the intervention communities. We increased this figure to 720 households to allow us to explore differences in effect size across communities. In addition, we enrolled the same number of households per community (180) in the two control communities, to arrive at a total sample size of 1080 households.

The main aim of the analysis was the comparison of changes from baseline to follow up between intervention and control sites. Statistical tests to compare the two study arms were not applied as the low number of clusters precluded this. Instead, we compared baseline and follow up separately for each community, and for intervention and control arm separately. For the between‐arm comparison of baseline and follow‐up socio‐economic characteristics we calculated difference‐in‐difference figures without confidence intervals to explore differential changes in these variables in intervention and control communities. Continuous outcomes were analysed using linear regression. Binary outcomes were analysed using binomial regression for risk/prevalence differences (link function: identity, distribution family binomial). Ordered categorical outcomes were analysed using ordered logistic regression with changes expressed as odds ratios. Fly counts were compared across categories using negative binomial regression resulting in rate ratios. Statistical analyses were done in STATA 14.

### Ethics approval

Ethics approval was obtained from the Research Ethical Committee of Allama Iqbal Open University and the Ethics Committee at the London School of Hygiene and Tropical Medicine (REC ID 15998). Informed consent for the questionnaire survey and fly trapping was obtained from the adult respondent of the questionnaire.

## RESULTS

Socioeconomic characteristics in intervention and control communities at baseline and follow up are shown in Table 1. Several indicators, that is, respondents' education, availability of a working fridge and drinking water access were more favourable in the control than in the intervention arm. As expected, control arm households on average reported a longer period of residence in the current location than intervention households. The difference‐in‐difference estimate indicates whether changes from baseline to follow‐up differed between intervention and control arms. The proportion of household heads not attending school decreased more in the control than the intervention arm, while the opposite was observed for respondents without any schooling. The proportion of households without a fridge decreased in the control arm but remained the same in the intervention arm. Access to tap water at home decreased strongly in the control arm but not in the intervention arm. Sewerage connection increased in the control arm but not in the intervention arm.

**TABLE 1:** Socio‐economic characteristics of intervention and control communities at baseline and follow up

	Intervention								Control				Total intervention		Total control		DiD
	Community 1 (1st batch)		Community 2 (1st batch)		Community 3 (2nd batch)		Community 4 (2nd batch)		Community 5		Community 6						
	BL	FU	BL	FU	BL	FU	BL	FU	BL	FU	BL	FU	BL	FU	BL	FU	
HH size (mean)	7.9	6.5	11.7	7.2	6.3	7.7	7.4	8.3	7.0	6.5	8.2	7.7	8.3	7.4	7.6	7.1	−0.4
Education level (HH head), %																	
Did not attend school	50.0	64.8	69.4	55.6	53.9	43.5	47.2	50.3	75.6	49.7	43.9	53.6	55.1	53.6	59.7	51.7	6.5
Some Primary	10.6	5.0	5.6	5.0	11.1	6.8	5.0	6.2	2.8	6.6	7.8	6.2	8.1	5.7	5.3	6.4	−3.5
Completed primary	6.7	3.9	0.6	0.6	2.8	1.7	6.7	1.7	3.9	3.3	4.4	2.2	4.2	2.0	4.2	2.8	−0.8
Some middle	6.1	7.7	5.6	7.2	5.6	7.3	3.9	8.4	1.1	8.2	8.3	8.4	5.3	7.7	4.7	8.3	−1.2
Completed middle	6.1	3.3	7.8	6.1	10.0	7.3	7.8	8.4	5.0	8.7	8.9	5.6	7.9	6.3	6.9	7.2	−1.9
Some secondary	3.3	4.4	1.1	5.6	3.3	3.4	3.9	5.6	2.8	4.4	4.4	6.7	2.9	4.7	3.6	5.5	−0.1
Completed secondary	11.1	7.1	8.3	12.8	8.9	9.0	16.7	3.9	7.8	3.8	6.7	9.5	11.3	8.2	7.2	6.6	−2.5
Some higher secondary	2.2	1.1	1.1	2.2	1.1	2.8	1.1	1.7	0.0	1.6	2.8	1.7	1.4	2.0	1.4	1.7	0.3
Completed higher secondary	2.8	2.2	0.0	1.7	3.3	1.7	7.8	2.8	1.1	0.0	7.8	1.1	3.5	2.1	4.4	0.6	2.4
Higher education	1.1	0.6	0.6	3.3	0.0	16.4	0.0	11.2	0.0	13.7	5.0	5.0	0.4	7.8	2.5	9.4	0.5
Education level (respondent), %																	
Did not attend school	52.2	53.3	55.0	45.0	41.1	31.1	29.4	44.7	32.8	44.3	32.2	31.8	44.4	43.6	32.5	38.1	−6.4
Some primary	10.0	3.3	8.9	8.3	9.4	13.0	6.1	8.9	8.9	8.7	7.2	6.7	8.6	8.4	8.1	7.7	0.2
Completed primary	7.2	5.5	0.6	0.0	6.1	1.7	7.8	1.7	7.8	1.6	7.8	2.8	5.4	2.2	7.8	2.2	2.4
Some middle	5.6	7.7	10.6	6.7	8.9	7.9	8.3	10.1	5.0	10.9	8.3	9.5	8.3	8.1	6.7	10.2	−3.7
Completed middle	5.0	7.1	8.3	6.7	11.1	7.3	10.6	5.6	8.3	6.0	12.8	11.2	8.8	6.7	10.6	8.6	−0.1
Some secondary	3.9	14.3	5.6	22.8	8.9	17.0	7.2	17.3	15.0	20.2	8.3	20.1	6.4	17.8	11.7	20.2	2.9
Completed secondary	12.8	3.3	7.8	0.0	7.8	7.3	16.7	0.0	15.0	1.1	4.4	0.6	11.3	2.7	9.7	0.8	0.3
Some higher secondary	1.7	2.8	2.2	6.7	1.7	5.1	5.0	1.7	0.0	3.8	8.3	7.8	2.6	4.0	4.2	5.8	−0.2
Completed higher secondary	1.7	1.7	0.0	2.8	5.0	4.0	8.9	7.8	7.2	1.1	5.6	3.4	3.9	4.0	6.4	2.2	4.3
Higher education	0.0	1.1	1.1	1.1	0.0	5.7	0.0	2.2	0.0	2.2	5.0	6.2	0.3	2.5	2.5	4.1	0.6
Fridge																	0
None	94.4	94.0	96.1	81.7	92.8	89.3	10.0	24.6	3.9	2.2	26.7	7.3	73.3	72.4	15.3	4.7	9.7
Yes–not working	3.9	6.0	3.3	9.4	1.7	1.7	2.2	2.8	0.0	2.7	5.6	1.1	2.8	5.0	2.8	1.9	3.1
Yes—working	1.7	0.0	0.6	8.9	5.6	9.0	87.8	72.6	96.1	95.1	67.8	91.6	23.9	22.6	81.9	93.4	−12.8
Number of years living at current residence (mean)	6.8	5.9	23.0	19.0	12.4	28.2	24.4	21.6	31.3	14.1	22.8	26.2	16.6	18.6	27.0	20.0	9
Drinking water source																	
Filter plan	7.2	4.4	3.9	19.4	71.1	84.8	58.3	26.8	73.3	82.0	24.4	70.4	35.1	33.6	48.9	76.2	−28.8
Public borehole	0.6	8.8	1.1	3.9	18.3	3.4	18.9	43.6	3.9	5.5	17.2	17.3	9.7	14.9	10.6	11.3	4.5
Private borehole owned by someone else	74.4	86.3	0.0	1.1	6.7	3.4	4.4	17.9	6.7	4.9	13.9	3.9	21.4	27.4	10.3	4.4	11.9
Borehole owned by HH	17.8	0.6	0.0	6.7	2.8	2.3	14.4	10.1	3.3	6.6	41.7	0.6	8.8	4.9	22.5	3.6	15
Supply water/Tape water to home	0.0	0.0	95.0	68.9	1.1	6.2	3.9	1.7	12.8	1.1	2.8	7.8	25.0	19.2	7.8	4.4	−2.4
Water for washing source																	0
Filter plan	3.3	1.7	0.6	12.8	0.6	5.7	3.3	2.8	1.1	14.8	2.2	20.7	1.9	5.7	1.7	17.7	−12.2
Public borehole	0.6	8.8	1.1	5.6	76.7	16.4	28.3	55.3	2.8	12.0	26.1	44.7	26.7	21.5	14.4	28.2	−19
Private borehole owned by someone else	77.2	87.9	0.0	3.3	7.8	17.0	15.0	25.1	15.0	12.6	16.1	14.0	25.0	33.6	15.6	13.3	10.9
Borehole owned by HH	18.9	0.6	0.0	1.1	6.1	10.7	45.0	10.6	7.2	48.6	50.0	0.6	17.5	5.7	28.6	24.9	−8.1
Supply water/Tape water to home	0.0	1.1	98.3	77.2	8.9	50.3	8.3	6.2	73.9	12.0	5.6	20.1	28.9	33.6	39.7	16.0	28.4
Water location																	
Piped to house	47.2	28.6	98.3	85.0	14.4	64.4	64.4	60.3	87.2	29.5	96.7	59.2	56.1	59.5	91.9	44.2	51.1
Using rubber pipe to someone else's tube	33.3	52.2	1.7	3.3	0.0	17.0	6.1	24.6	6.7	9.3	2.8	16.8	10.3	24.4	4.7	13.0	5.8
outside house, within 10 min walk	14.4	18.1	0.0	6.1	80.6	18.1	22.8	15.1	4.4	56.3	0.6	20.7	29.4	14.4	2.5	38.7	−51.2
Outside house, more than 10 min walk	5.0	1.1	0.0	5.6	5.0	0.0	6.7	0.0	1.7	4.9	0.0	3.4	4.2	1.7	0.8	4.1	−5.8
Do not know	0.0	0.0	0.0	0.0	0.0	0.6	0.0	0.0	0.0	0.0	0.0	0.0	0.0	0.1	0.0	0.0	0.1
Toilet ownership																	
No	0.6	0.0	0.0	0.0	0.0	0.0	0.0	0.0	1.1	0.0	0.0	0.0	0.1	0.0	0.6	0.0	0.5
Pour flush to pit	0.6	0.6	0.0	0.0	0.0	0.0	0.6	0.0	0.0	0.0	1.1	0.0	0.3	0.1	0.6	0.0	0.4
Pour flush to septic tank	0.0	0.0	0.0	0.0	0.0	0.0	0.0	0.0	0.0	0.0	20.0	0.0	0.0	0.0	10.0	0.0	10
Pour flush to river	0.0	0.0	6.1	2.2	1.7	0.0	1.7	0.0	2.2	1.6	0.6	1.1	2.4	0.6	1.4	1.4	−1.8
Pour flush to sewerage	98.9	99.5	93.3	97.8	98.3	98.9	97.8	100.0	96.1	98.4	78.3	98.9	97.1	99.0	87.2	98.6	−9.5
Composting toilet	0.0	0.0	0.6	0.0	0.0	0.0	0.0	0.0	0.6	0.0	0.0	0.0	0.1	0.0	0.3	0.0	0.2
Do not know	0.0	0.0	0.0	0.0	0.0	1.1	0.0	0.0	0.0	0.0	0.0	0.0	0.0	0.3	0.0	0.0	0.3

Abbreviations: BL, baseline; DiD, difference‐in‐difference; FU, follow up.

Flooding of drains decreased in both arms, while flooding of land remained constant (Table 2). Flooding of houses decreased slightly in the intervention arm and more so in the control arm. Exposure to smoke from waste burning (inside and outside compound), as well as the reported presence of pests (cockroaches, rats) inside and outside compound all decreased to a similar extent in intervention and control. Overall, there was no evidence that these exposures decreased more in the first two communities (Batch 1) than in the second intervention batch. However, a strong reduction in the number of days of smoke exposure (inside and outside of compound) was observed in Community 1.

**TABLE 2:** Environmental conditions in intervention and control communities at baseline and follow up

	Intervention							Control					Total intervention			Total control		
	Community 1 (1st batch)		Community 2 (1st batch)		Community 3 (2nd batch)		Community 4 (2nd batch)		Community 5		Community 6							
	BL	FU	BL	FU	BL	FU	BL	FU	BL	FU	BL	FU	BL	FU	OR/Diff (95%CI)	BL	FU	OR/Diff (95%CI)
Drain flooding (%)															OR 0.6 (0.4, 0.8)			OR 0.3 (0.2, 0.5)
No	91.7	91.8	92.8	85.6	65.0	90.4	78.3	85.5	56.1	90.7	92.8	88.3	81.9	88.3		74.4	89.5	
Yes, less than once per year	0.0	3.3	7.2	0.6	0.0	0.6	10.0	0.0	0.6	0.0	0.6	1.1	4.3	1.1		0.6	0.6	
Yes, once a year	2.2	0.6	0.0	0.6	0.6	2.3	5.6	1.1	2.2	0.0	0.0	1.1	2.1	1.1		1.1	0.6	
Yes, twice a year	0.0	2.2	0.0	3.9	5.0	1.1	1.7	1.7	1.7	0.6	2.2	0.0	1.7	2.2		1.9	0.3	
Yes, more than a twice a year	6.1	2.2	0.0	9.4	29.4	5.7	4.4	11.7	39.4	8.7	4.4	9.5	10.0	7.2		21.9	9.1	
Land flooding (%)															OR 0.9 (0.7, 1.2)			OR 0.9 (0.6, 1.5)
No	93.9	80.2	88.9	86.1	65.0	89.8	86.7	84.4	90.0	89.1	89.4	92.7	83.6	85.1		89.7	90.9	
Yes, less than once per year	0.0	2.8	10.0	1.7	0.6	2.8	9.4	0.0	0.6	1.6	0.6	0.6	5.0	1.8		0.6	1.1	
Yes, once a year	1.1	0.6	1.1	0.6	0.0	2.3	1.7	0.6	5.0	0.0	3.9	1.1	1.0	1.0		4.4	0.6	
Yes, twice a year	1.1	1.1	0.0	3.3	1.7	1.1	1.1	1.7	1.1	0.0	0.6	1.1	1.0	1.8		0.8	0.6	
Yes, more than a twice a year	3.9	15.4	0.0	8.3	32.8	4.0	1.1	13.4	3.3	9.3	5.6	4.5	9.4	10.3		4.4	6.9	
House flooding (%)															OR 0.7 (0.4, 1.0)			OR 0.2 (0.1, 0.5)
No	96.1	92.3	92.8	96.1	92.2	93.2	85.6	95.5	91.7	97.8	90.0	97.8	91.7	94.3		90.8	97.8	
Yes, less than once per year	1.1	1.7	6.7	1.1	0.6	2.8	9.4	1.7	0.0	1.1	1.1	0.0	4.4	1.8		0.6	0.6	
Yes, once a year	0.6	1.1	0.6	0.6	1.1	2.3	1.7	0.0	3.3	0.0	3.3	1.1	1.0	1.0		3.3	0.6	
Yes, twice a year	0.0	0.0	0.0	1.1	1.1	0.6	1.7	0.0	2.2	0.0	0.6	0.6	0.7	0.4		1.4	0.3	
Yes, more than a twice a year	2.2	5.0	0.0	1.1	5.0	1.1	1.7	2.8	2.8	1.1	5.0	0.6	2.2	2.5		3.9	0.8	
Waste burning smoke inside compound (days per week)	1.4	0.5	0.2	0.2	0.3	0.2	0.3	0.3	0.6	0.3	0.4	0.1	0.5	0.3	Diff −0.2 (−0.3, −0.1)	0.5	0.2	Diff −0.3 (−0.4, −0.2)
Smoke in community (days per week)	1.2	0.4	0.2	0.2	0.3	0.3	0.3	0.3	0.3	0.2	0.2	0.2	0.5	0.3	Diff −0.2 (−0.3, −0.1)	0.2	0.2	Diff 0.0 (−0.2, 0.1)
Seen rats in house (days per week)	1.4	1.8	0.7	0.9	1.4	0.5	1.6	0.7	0.6	1.0	2.0	0.8	1.3	1.0	Diff −0.3 (−0.5, −0.1)	1.3	0.9	Diff −0.4 (−0.7, 0.0)
Seen rats in community (days per week)	0.8	1.6	0.7	1.1	1.5	0.7	2.2	0.7	1.6	1.2	1.3	1.1	1.3	1.0	Diff −0.3 (−0.5, −0.3)	1.5	1.1	Diff −0.3 (−0.6, 0.0)
Seen cockroaches in house (days per week)	0.2	0.3	0.6	0.6	0.7	0.3	1.1	0.3	0.4	0.3	1.4	0.5	0.6	0.4	Diff −0.2 (−0.4,‐0.1)	0.9	0.4	Diff −0.5 (−0.8, −0.3)
Seen cockroaches in community (days per week)	0.0	0.2	0.7	0.4	0.6	0.3	1.2	0.2	0.7	0.1	1.1	0.3	0.6	0.3	Diff −0.3 (−0.5, −0.2)	0.9	0.2	Diff −0.7 (−0.9, −0.5)

Abbreviations: BL, baseline; Diff, difference in prevalence from binomial regression (identity link, binomial family); FU, follow up; OR, odds ratio from ordered logistic regression with the first category as base.

In the first intervention batch there were strong increases in ‘waste being perceived as no problem at all’, ‘perceived improvement in waste management over the past year’, and ‘waste being removed from the compound while not being dumped outside’. Similar improvements were not observed in the other two intervention communities (Table 3). Considerable improvements in these items were also observed in the control communities, mainly as a result of the practice of bringing waste to collection points being replaced by doorstep collection. Community 2 (first batch), which previously had the highest prevalence of burning waste as main means for dealing with household waste, had no household reporting this practice as their main means after the intervention. Overall, however, there were no consistent reductions in any burning of waste across intervention and control communities. In both study arms, ‘once daily’ became the most common frequency of waste collection. Storing waste inside the house increased strongly in the intervention arm, with a lesser increase also observed in the control arm. In both arms, storage using a closed container (observed) increased almost uniformly across intervention and control arms. In both arms, but especially in the first intervention batch, reported household‐level responsibility for waste management shifted from being largely men towards a situation where everyone was responsible.

**TABLE 3:** Waste disposal practices in intervention and control communities at baseline and follow up

	Intervention								Control				Total intervention			Total control		
	Community 1 (1st batch)		Community 2 (1st batch)		Community 3 (2nd batch)		Community 4 (2nd batch)		Community 5		Community 6							
	BL	FU	BL	FU	BL	FU	BL	FU	BL	FU	BL	FU	BL	FU	OR/Diff (95% CI)	BL	FU	OR/Diff (95% CI)
Waste perceived as problem (%)															OR 0.2 (0.1, 0.2)			OR 0.04 (0.03, 0.07)
No problem at all	1.7	80.8	0.0	83.3	16.1	13.1	43.3	10.6	44.4	78.7	2.8	55.3	15.3	47.4		23.6	67.1	
Small problem	19.4	15.4	43.9	13.3	18.9	48.0	37.2	54.8	50.6	18.6	0.6	32.4	29.9	32.7		25.6	25.4	
Big problem	50.6	3.9	38.3	3.3	45.0	36.0	17.2	29.6	5.0	1.6	59.4	10.6	37.8	18.0		32.2	6.1	
Very big problem	28.3	0.0	17.8	0.0	20.0	2.9	2.2	5.0	0.0	1.1	37.2	1.7	17.1	2.0		18.6	1.4	
In last year waste management improved (%)															OR 0.20 (0.16, 0.24)			OR 0.21 (0.16, 0.29)
A lot better	0.6	79.1	0.0	80.0	1.7	0.6	13.3	1.7	45.0	64.5	3.9	46.9	3.9	40.7		24.4	55.8	
A bit better	50.0	15.9	13.3	15.0	1.7	20.3	62.2	16.2	42.2	26.2	10.6	21.2	31.8	16.9		26.4	23.8	
No change/do not know	48.9	2.8	25.6	2.2	90.0	74.0	21.7	79.9	11.1	8.2	85.0	30.2	46.5	39.4		48.1	19.1	
A bit worse	0.6	1.1	59.4	1.7	6.7	2.3	2.8	1.7	1.7	0.0	0.0	1.7	17.4	1.7		0.8	0.8	
A lot worse	0.0	1.1	1.7	1.1	0.0	2.8	0.0	0.6	0.0	1.1	0.6	0.0	0.4	1.4		0.3	0.6	
Main waste disposal practice (%)															OR 3.3 (2.7, 4.0)			OR 11.2 (8.1, 15.6)
Buried by HH	0.0	0.6	2.2	0.0	0.0	0.6	0.0	0.0	1.1	0.0	1.1	0.0	0.6	0.3		1.1	0.0	
Burnt by HH	1.1	0.0	5.6	0.0	1.7	1.7	1.1	1.7	1.1	0.0	0.6	0.0	2.4	0.8		0.8	0.0	
Dumped in compound	17.2	1.7	1.1	2.2	18.3	13.6	18.3	36.9	24.4	2.2	64.4	11.2	13.8	13.5		44.4	6.6	
Taken out of compound and thrown by river/open ground	48.9	1.7	56.7	0.0	0.0	29.9	0.0	41.9	0.0	3.8	15.0	19.0	26.4	18.3		7.5	11.3	
Taken out of compound and disposed into container/collection point	8.3	3.9	34.4	13.9	77.8	26.6	67.8	12.9	39.4	9.3	10.6	12.3	47.1	14.2		25.0	10.8	
Is collected and removed from the doorstep	24.4	92.3	0.0	83.9	2.2	27.1	12.8	6.7	33.9	84.7	8.3	57.5	9.9	52.8		21.1	71.3	
Do not know	0.0	0.0	0.0	0.0	0.0	0.6	0.0	0.0	0.0	0.0	0.0	0.0	0.0	0.1		0.0	0.0	
Burnt waste in last week (%)	3.9	0.6	4.4	12.8	3.3	5.8	21.7	1.1	3.3	4.9	2.8	6.2	8.3	5.0	Diff −2.6 (−4.7, −0.5)	3.1	5.5	Diff 2.5 (−0.5, 5.4)
Frequency of waste disposal (%)															OR 1.5 (1.1, 2.1)			OR 3.1 (2.0, 5.0)
More than once a day	8.3	1.1	23.3	1.1	3.3	7.4	8.3	9.5	2.2	2.2	32.8	2.2	10.8	4.6		17.5	2.2	
Once a day	82.8	97.8	71.7	97.8	95.0	76.6	83.3	84.4	88.3	96.7	66.1	92.7	83.2	89.1		77.2	94.8	
More than once a week, not every day	7.8	0.6	1.7	0.6	1.1	6.9	7.8	6.2	9.4	0.0	0.0	3.9	4.6	3.6		4.7	1.9	
Once a week	0.6	0.0	1.1	0.0	0.6	1.1	0.6	0.0	0.0	0.0	1.1	0.0	0.7	0.4		0.6	0.0	
Less than once a week	0.6	0.6	2.2	0.6	0.0	1.1	0.0	0.0	0.0	1.1	0.0	1.1	0.7	0.6		0.0	1.1	
Do not know	0.0	0.0	0.0	0.0	0.0	6.9	0.0	0.0	0.0	0.0	0.0	0.0	0.0	1.7		0.0	0.0	
Frequency of removal at collection site (%)															OR 0.62 (0.48, 0.81)			OR 0.85 (0.59, 1.21)
More than once a day	12.8	0.6	24.4	8.3	1.1	19.2	3.9	20.7	1.7	2.2	26.1	8.9	10.6	12.1		13.9	5.5	
Once a day	58.9	97.3	71.7	86.1	96.7	65.5	77.8	77.1	68.9	94.5	70.0	84.4	76.3	81.6		69.4	89.5	
More than once a week, not every day	7.2	1.1	2.2	0.6	1.1	4.0	8.3	2.2	27.8	0.6	0.0	2.2	4.7	2.0		13.9	1.4	
Once a week	0.0	0.0	0.6	0.6	0.6	0.6	3.3	0.0	1.7	0.6	0.6	1.1	1.1	0.3		1.1	0.8	
Less than once a week	21.1	1.1	1.1	4.4	0.6	4.0	6.7	0.0	0.0	2.2	0.0	3.4	7.4	2.4		0.0	2.8	
No collection	0.0	0.0	0.0	0.0	0.0	6.8	0.0	0.0	0.0	0.0	3.3	0.0	0.0	1.7		1.7	0.0	
Do not know																		
Person responsible in HH (%)																		
Adult women	30.6	38.5	35.6	27.8	18.9	22.6	36.1	26.8	25.0	33.9	33.3	30.2	30.3	29.0	Diff −1.1 (−5.7, 3.6)	29.2	32.0	Diff 3.0 (−3.7, 9.7)
Adult men	36.1	12.6	39.4	16.1	21.1	35.0	35.0	22.9	30.0	11.5	16.7	9.5	32.9	21.6	Diff −12.0 (−16.5, −7.4)	23.3	10.5	Diff −12.0 (−17.4, −6.7)
Boys	3.9	5.0	10.6	3.9	10.6	7.3	7.8	6.7	5.0	2.7	5.0	3.9	8.2	5.7	Diff −2.1 (−4.7, 0.5)	5.0	3.3	Diff −1.7 (−4.6, 1.2)
Girls	2.8	1.1	3.3	3.9	1.7	2.8	2.8	1.7	0.0	1.1	0.6	0.6	2.6	2.4	Diff −0.4 (−2.0, 1.1)	0.3	0.8	Diff 0.7 (−0.3, 1.8)
Everyone	26.7	42.9	11.1	48.3	47.8	31.1	18.3	41.9	40.0	50.8	44.4	55.9	26.0	41.1	Diff 16.5 (11.8, 21.3)	42.2	53.3	Diff 11.1 (3.9, 18.4)
Do not know	0.0	0.0	0.0	0.0	0.0	1.1	0.0	0.0	0.0	0.0	0.0	0.0	0.0	0.3	NA	0.0	0.0	NA
																		
Storage of household waste before disposing in last week? (%)															OR 0.65 (0.54, 0.79)			OR 0.85 (0.65, 1.11)
In the house	31.1	48.9	15.0	53.3	5.0	36.2	23.9	60.3	22.8	46.5	38.3	40.2	18.8	49.7		30.6	43.4	
In the yard/compound	45.6	9.3	82.2	5.6	42.8	16.4	33.9	8.9	47.8	13.7	10.0	15.6	51.1	10.0		28.9	14.6	
Outside yard / compound	22.8	40.1	2.8	30.6	43.9	28.3	28.3	15.6	23.3	34.4	46.7	27.4	24.4	28.7		35.0	30.9	
Not stored	0.6	1.7	0.0	10.6	8.3	19.2	13.9	15.1	6.1	5.5	5.0	16.8	5.7	11.6		5.6	11.1	
Household waste stored in a covered container (observed, %)	12.8	68.1	27.2	52.3	11.1	71.5	40.0	72.6	18.9	77.8	33.3	65.2	26.1	67.7	Diff 43.4 (38.8, 48.0)	22.8	65.9	Diff 45.0 (38.4, 51.7)

Abbreviations: BL, baseline; Diff, difference in prevalence from binomial regression (identity link, binomial family); FU, follow up; OR, odds ratio from ordered logistic regression with the first category as base.

Availability and use of waste collection services increased in all communities except community 4 (second intervention batch, Table 4). The improvement was particularly strong in the first intervention batch. Satisfaction with the service improved in the first intervention batch and in the control communities but not in the second intervention batch communities. Table 5 suggests a strong increase in the proportion of households that disposed of disposable nappies/diapers in the garbage and a decrease in those disposing of child faeces in the toilet.

**TABLE 4:** Waste collection service availability in intervention and control communities at baseline and follow up

	Intervention								Control				Total intervention			Total control		
	Community 1 (1st batch)		Community 2 (1st batch)		Community 3 (2nd batch)		Community 4 (2nd batch)		Community 5		Community 6							
	BL	FU	BL	FU	BL	FU	BL	FU	BL	FU	BL	FU	BL	FU	OR/Diff (95%CI)	BL	FU	OR/Diff (95%CI)
Waste collection available at doorstep (%)	0.6	94.5	0.0	86.1	3.9	24.9	29.4	5.0	57.2	80.3	6.7	73.2	8.5	52.9	Diff 44.3 (40.4, 48.2)	31.9	76.8	Diff 44.6 (38.4, 51.0)
Uses waste collection service (%)	0.0	92.3	0.0	82.7	5.0	15.8	15.0	5.6	37.2	76.0	5.0	57.9	5.0	49.4	Diff 44.2 (40.6, 47.8)	21.1	67.0	Diff 46.5 (40.5, 52.6)
Why not using collection service (*n*)	**0**	**13**	**0**	**67**	**171**	**11**	**153**	**2**	**113**	**24**	**3**	**92**	**324**	**93**		**116**	**116**	
No money	—	30.8	—	71.6	2.3	45.5	0.7	0.0	0.0	16.7	0.0	23.9	1.5	61.3	Diff 36.1 (22.5, 49.7)	0.0	22.4	Diff 17.6 (5.3, 29.8)
They are not coming here	—	23.1	—	9.0	59.7	18.2	33.3	50.0	0.0	20.8	33.3	6.5	47.2	12.9	Diff −32.1 (−57.7, −6.6)	0.9	9.5	Diff 14.7 (5.8, 23.7)
They are not reliable	—	30.8	—	0.0	6.4	27.3	13.1	0.0	0.0	4.2	0.0	3.3	9.6	7.5	Diff 15.4 (−0.4, 31.2)	0.0	3.5	Diff 4.1 (−1.3, 9.4)
Other	—	15.4	—	19.4	31.6	9.1	52.9	50.0	100.0	58.3	66.7	66.3	41.7	18.3	Diff −19.4 (−45,4, 6.6)	99.1	64.7	Diff −36.4 (−50.7, −22.1)
Satisfaction with service (%)															OR 2.2 (0.8, 6.1)			OR 0.1 (0.1, 0.2)
Very satisfied	NA	93.6	NA	93.9	25.0	8.7	64.0	41.7	49.2	86.5	0.0	84.8	54.6	86.8		41.3	85.8	
Fairly satisfied	NA	5.2	NA	3.6	62.5	91.3	36.0	33.3	46.0	11.0	33.3	9.8	42.4	10.8		44.0	10.6	
Neither satisfied nor dissatisfied	NA	0.0	NA	0.0	12.5	0.0	0.0	25.0	4.8	1.8	66.7	5.4	3.0	0.8		14.7	3.3	
Fairly dissatisfied	NA	1.2	NA	0.6	0.0	0.0	0.0	0.0	0.0	0.0	0.0	0.0	0.0	0.8		0.0	0.0	
Very dissatisfied	NA	0.0	NA	1.8	0.0	0.0	0.0	0.0	0.0	0.6	0.0	0.0	0.0	0.8		0.0	0.4	

Abbreviations: BL, baseline; Diff, difference in prevalence from binomial regression (identity link, binomial family); FU, follow up; OR, odds ratio from ordered logistic regression with the first category as base.

**TABLE 5:** Child faeces disposal in intervention and control communities at baseline and follow up

	Intervention							Control					Total intervention			Total control		
	Community 1 (1st batch)		Community 2 (1st batch)		Community 3 (2nd batch)		Community 4 (2nd batch)		Community 5		Community 6							
	BL	FU	BL	FU	BL	FU	BL	FU	BL	FU	BL	FU	BL	FU	OR/Diff (95%CI)	BL	FU	OR/Diff (95%CI)
Child faeces disposal	**n= 109**	**n= 83**	**n= 123**	**n= 77**	**n= 79**	**n= 66**	**n= 83**	**n= 70**	**n= 92**	**n= 75**	**n= 115**	**n= 83**	**n= 394**	**n= 300**		**n= 207**	**n= 159**	
Left where they were	0.9	0.0	0.0	0.0	0.0	0.0	0.0	2.7	0.0	0.0	0.0	1.2	0.3	0.7	Diff 0.4 (−0.6, 1.4)	0.0	0.6	Diff 0.6 (−0.4, 1.7)
Thrown out of compound	0.0	1.2	0.0	0.0	0.0	0.0	3.6	2.7	0.0	0.0	3.5	2.4	0.8	1.0	Diff 0.1 (−1.2, 1.5)	1.9	1.3	Diff −0.6 (−3.2, 2.1)
Thrown in the drain	0.0	0.0	0.0	11.7	0.0	0.0	0.0	0.0	0.0	1.3	0.0	1.2	0.0	3.0	Diff 3.3 (1.6, 4.9)	0.0	1.3	Diff 1.3 (−0.3, 2.9)
Thrown in garbage	3.7	97.6	0.8	88.3	53.2	100.0	77.1	94.6	98.9	98.7	67.0	95.2	28.2	95.0	Diff 64.5 (59.7, 69.3)	81.2	96.9	Diff 15.0 (8.8, 21.3)
Thrown in latrine	95.4	1.2	99.2	0.0	46.8	0.0	19.3	0.0	1.1	0.0	29.6	0.0	70.8	0.3	Diff −68.1 (−72.6, −63.9)	16.9	0.0	Diff −16.4 (−22.0, −10.7)

Abbreviations: BL, baseline; Diff, difference in prevalence from binomial regression (identity link, binomial family); FU, follow up; OR, odds ratio from ordered logistic regression with the first category as base.

**FIGURE 2:**
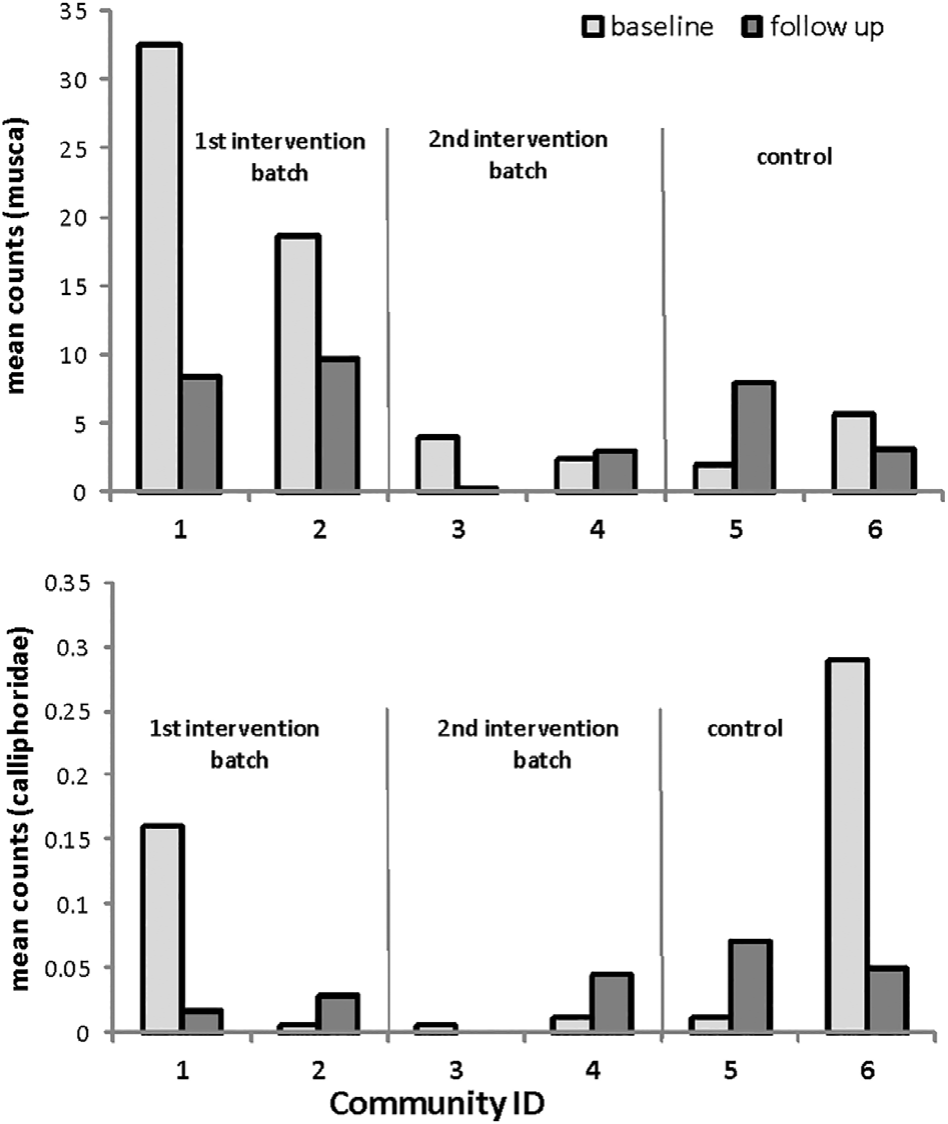
(a) Musca fly counts and (b) calliphoridae fly counts in intervention and control communities at baseline and follow up

Fly counts (*muscae*) decreased by 64% in the intervention arm (rate ratio 0.36, 95% CI 0.29, 0.44), while they increased by 52% in the control arm (rate ratio 1.52, 95% CI 1.07, 2.16). In the intervention arm, the reduction in counts was particularly pronounced in the first intervention batch, and absent in intervention Community 4 (Figure 2a). *Calliphoridae* counts were generally low with little statistical support for relevant changes in counts from baseline to follow up (intervention rate ratio 0.79, 95% CI 0.27, 2.30, control rate ratio 0.83, 95% CI 0.15, 4.66, Figure 2b).

**FIGURE 3:**
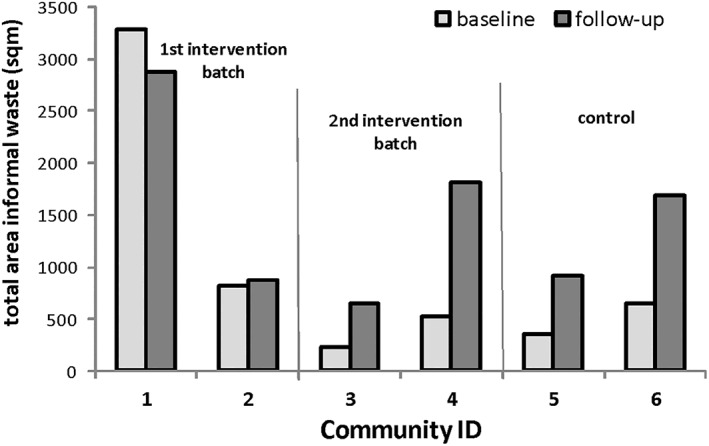
Area in square meters covered by informal waste disposal sites in intervention and control communities at baseline and follow up

The community‐level environmental survey found that the area covered by informal waste remained approximately constant in the first intervention batch but increased in the second intervention batch and in the control communities (Figure 3).

## DISCUSSION

This study suggests that implementing a low‐cost improved waste collection service with centralised waste processing in urban low‐income communities can improve waste management at household level and reduce the exposure to synanthropic flies. However, the study shows that reach of the intervention differed greatly among communities, suggesting challenges with implementation and community involvement faced by such programmes. The study further highlights changes in disposal practices of child faeces after the intervention that may pose occupational hazards for waste segregation staff. Finally, the intervention had little effect on informal waste sites prevalent in the study communities, despite high adoption of the waste collection service.

The study evaluated a real‐life waste disposal intervention that targeted underserved communities which nevertheless differed greatly in size, socio‐economic status and community cohesion. The first two communities enrolled (Batch 1) were marked by poor access to public services as they were not recognised as legal settlements by government authorities. However, community cohesion and prior absence of services appear to have facilitated adoption of the programme. Most households reported an improvement in waste management and satisfaction with services after the intervention. Perhaps as a result, these communities experienced a strong reduction in fly counts at household level.

Such improvements were not consistently observed in the other two intervention communities. Both second‐batch communities had better prior access to public services due to some recognition of their legal status by authorities. However, the team implementing the intervention reported a poor response from community leaders and lack of interest in the intervention by some households that seemed to have reduced acceptability and reach of the intervention in these two communities.

Most communities experienced an increase in the amount of waste disposed of at informal waste sites, except the Batch 1 intervention communities where adoption of services was highest. These two communities were also somewhat isolated, whereas the other communities were surrounded by built‐up urban areas. Possibly, the waste deposited in the Batch 1 communities largely came from within the communities themselves, while in the other communities, the dump sites may have been used by people or businesses from other communities. Further research might investigate the users and uses of informal waste sites to understand the extent to which they are an alternative or an addition to the use of doorstep collection services and the reasons underlying this. Future interventions might try combining behaviour change communication with initial removal of waste sites as a means of shifting social norms around waste disposal.

The effect of the intervention on reducing the burning of waste and its associated health effects [14] was inconsistent. Further research needs to be performed to identify reasons for the ongoing use of this disposal method, which could, for example, be due to convenience or perhaps to fire being perceived as a way of purifying waste.

This study did not attempt to estimate potentially negative effects of introducing systematic waste collection on the livelihood of existing waste pickers and scrap dealers. These people were intended to be included in the intervention by giving them a role as middlemen in the sale of higher‐value recyclables such as plastic and metal. The intervention removed the need for them to conduct house‐to‐house visits and pay for the recyclables at the doorstep and was reportedly well received by these groups. By far the largest volume of waste was organic waste which was previously not dealt with at all by the informal waste sector. Some income loss for households who previously sold recyclables to waste pickers may have occurred, but the benefit of having waste removed in its entirety seemed to have offset this disadvantage for most households. Future studies should aim at providing a more comprehensive picture of the effects of introducing waste disposal services in low‐income settings where waste is not only seen as a nuisance or environmental hazard but also as an important source of income and employment.

Improvements in waste collection services and satisfaction with services were not only observed in the Batch 1 intervention communities but (to a lesser extent) also in the two control communities. The control communities were a sub‐optimal choice as controls. Both had been established several decades ago (pre‐dating the intervention communities), benefitted from a recognised legal status, and enjoyed the best access to public services among study communities. For these reasons, they were not prioritised as target communities for the intervention. Efforts to identify more suitable control communities failed, making the comparison with the intervention communities difficult.

The control communities appear to have experienced improvements in waste management such as doorstep collection service and storage of waste in closed containers in the house or compound prior to collection. The recruitment of these control communities still proved worthwhile. The data suggested that highly underserved communities in which the intervention was successfully implemented (i.e., the first batch of communities) could “catch up” with the more established communities in terms of reducing perceived problems with waste collection, increasing satisfaction with services and reducing fly counts and (to some extent) the area of waste present in the public domain.

In addition to the intervention not being randomly allocated and the lack of suitable control communities, the study was limited by the small number of communities allocated to intervention and control arms. The waste disposal programme was implemented in only four communities at this stage, even though a similar model is being used in projects with communities elsewhere in Pakistan. The small number of clusters made statistical comparison across arms difficult, allowing only for a before‐and‐after comparison within intervention and control arms, and for each community separately. Furthermore, the two surveys were conducted as cross‐sectional studies rather than revisiting each household enrolled at baseline.

Comparison of socio‐economic characteristics between the two surveys revealed changes in several variables from baseline to follow up. Sampling procedures were the same at baseline and follow up but sampling variability or unknown biases in selecting households are likely to have influenced the composition of the study population and, possibly, the results.

Most outcomes were based on self‐report by the household respondent. We used a simple method to estimate fly counts which had been validated in a similar setting [12]. Because of the great variety in building and kitchen set‐ups among participating households, standardisation of placing the fly traps proved difficult. Assessing the amount of waste disposed of at informal waste sites in the public domain within the communities was also a challenge. Much of this waste was disposed of on uneven surfaces such as riversides and other slopes, making it difficult to estimate the volume. Due to government regulations, we were unable to use GPS to measure the size of waste sites. Instead, we used measuring tapes, which was cumbersome, especially in cases where the waste sites were of uneven shape. Overall, the study highlights methodological challenges in measuring the effect of a waste management intervention in a realistic setting, which may be why most previous studies have been largely observational (e.g., [6, 7, 9, 15, 16, 17]).

To conclude, the study shows that a centralised, low‐cost waste collection service can positively impact on waste disposal practices in urban low‐income communities and reduce exposure to health risks such as synanthropic flies. The study highlights methodological challenges in assessing the effect of improved waste disposal practices on health and wellbeing in low‐income settings.

## FUNDING INFORMATION

The study was funded by Tearfund.
